# Functional polarization of tumor-associated macrophages dictated by metabolic reprogramming

**DOI:** 10.1186/s13046-023-02832-9

**Published:** 2023-09-23

**Authors:** Wentao Zeng, Fei Li, Shikai Jin, Ping-Chih Ho, Pu-Ste Liu, Xin Xie

**Affiliations:** 1 School of Life and Environmental Sciences, Shaoxing University, Shaoxing, 312000 Zhejiang China; 2 Department of Fundamental Oncology, Faculty of Biology and Medicine, University of Lausanne, Lausanne, Switzerland; 3 Ludwig Lausanne Branch, Lausanne, Switzerland; 4 Institute of Cellular and System Medicine, National Health Research Institute, Miaoli, Taiwan, ROC

**Keywords:** TAMs, TME, Metabolic reprogramming, Polarization, Signaling pathways

## Abstract

Macrophages are highly plastic in different tissues and can differentiate into functional subpopulations under different stimuli. Tumor-associated macrophages (TAMs) are one of the most important innate immune cells implicated in the establishment of an immunosuppressive tumor microenvironment (TME). Recent evidence pinpoints the critical role of metabolic reprogramming in dictating pro-tumorigenic functions of TAMs. Both tumor cells and macrophages undergo metabolic reprogramming to meet energy demands in the TME. Understanding the metabolic rewiring in TAMs can shed light on immune escape mechanisms and provide insights into repolarizing TAMs towards anti-tumorigenic function. Here, we discuss how metabolism impinges on the functional divergence of macrophages and its relevance to macrophage polarization in the TME.

## Background

Tissue-resident macrophages have been recognized as integral components in different organs, and their functional polarization contributes to localized immune responses, tissue repair and homeostasis [1]. Macrophage-mediated extracellular matrix remodeling and angiogenesis participate in normal physiological development [2, 3]. Liver macrophages (also known as Kupffer cells) function to scavenge products derived from iron metabolism and intestinal derivatives [4]. Pulmonary macrophages are involved in the immune defense against invading pathogens as well as the maintenance of alveolar microenvironment by clearing pollutant and surfactant [5]. Perturbation of macrophage function is implicated in pathophysiological conditions such as metabolic disorders (obesity and arteriosclerosis), chronic inflammation (colitis and multiple sclerosis) and cancer progression [6–8].

Macrophage infiltration in the tumor microenvironment (TME) is a common determinant of the immunosuppression in different tumors [9]. TME represents a unique milieu for complex interactions between cancerous cells and immune cells. TME is characterized by a low-pH, hypoxic and sugar-deficient, and the lactic acid, lipids and cytokines-enriched milieu where the reprogramming of tumor-associated macrophages (TAMs) towards pro-tumorigenic phenotype is favored [10]. TAMs can not be simply defined by M1/M2 macrophage dichotomy since they possess mixed features of M1 and M2 macrophages [11, 12]. The expression of special receptor tyrosine kinases such as Tyro3, Axl, and MerTK has been reported in TAMs, and these receptors are implicated in the interaction with tumor cells, macrophage polarization and efferocytosis [13]. TAMs secrete a variety of factors, such as vascular endothelial growth factor (VEGF), to promote neo-vascularization and the metastasis of cancer cells [14]. Tumor-infiltrating macrophages also serve as the main source of anti-inflammatory cytokine IL-10 to establish an immunosuppressive TME [15]. There exist different macrophage subpopulations in a state between M1- and M2-type in the TME, which complicates the functional diversity of TAMs [16]. Nevertheless, recent evidence pinpoints the critical roles of metabolic reprogramming in dictating functional specification of TAMs [17]. Understanding the metabolic rewiring in TAMs provides opportunities to repolarize TAMs towards anti-tumorigenic function.

In this review, we summarize different signaling pathways involved in the metabolic rewiring of macrophage polarization, delineate the metabolic pattern of TAMs, and highlight TME-derived metabolites that regulate the functional polarization of TAMs. We also discuss recent developments in employing metabolic reprogramming to repolarize TAMs for anti-cancer purpose.

## Metabolic pattern of TAMs

Macrophages are one of the most predominant immune cell types in the TME, where TAMs can be reprogrammed into pro-tumorigenic phenotype to facilitate tumor progression or anti-tumorigenic phenotype to exert tumoricidal function. Although TAMs are predominantly polarized into M2-like pro-tumorigenic state in the TME, the high degree of macrophage plasticity allows re-directing TAMs into M1-like tumor-suppressive state. Distinct metabolic profiles underpin the functional specialization of macrophages in the TME [18]. Understanding the specific metabolic patterns of M1 and M2 polarization state is crucial for the metabolic manipulation of TAM activity in the TME.

### Glycolysis and pentose phosphate pathway

**Fig. 1:**
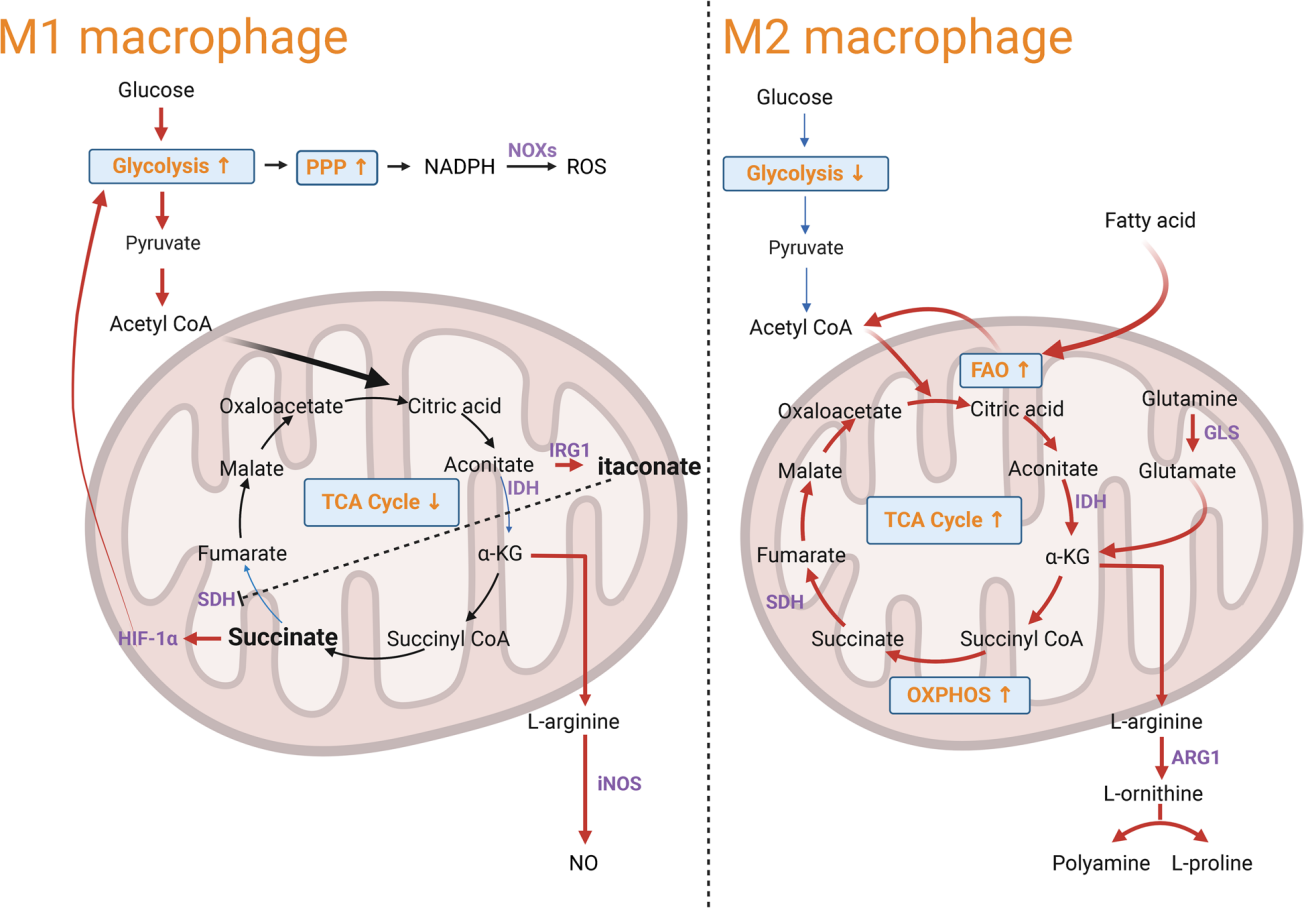
Distinct metabolic patterns of M1 and M2 macrophages. In M1 macrophages high gycolytic flux and the shunting of intermediates to PPP favor ROS production. The TCA cycle is truncated due to the expression of IRG1 and the impaired IDH activity. Itaconate production inhibits SDH and succinate accumulation further stabilizes HIF-1α to strengthen glycolysis. M2 macrophages reply on β-oxidation of fatty acids and glutaminolysis to drive TCA cycle. The production of polyamines and proline from L-arginine facilitate tumorigenesis. Abbreviations: α-KG, α-ketoglutarate; ARG1, arginase 1; FAO, fatty acid oxidation; GLS, glutaminase; HIF-1α, hypoxia inducible factor-1α; IDH, isocitrate dehydrogenase; iNOS, inducible nitric oxide synthase; IRG1, aconitate decarboxylase 1; NO, nitric oxide; NOXs, NADPH oxidases; PPP, pentose phosphate pathway; ROS, reactive oxygen species; SDH, succinate dehydrogenase; TCA, tricarboxylic acid (Created with BioRender.com)

One of the hallmarks in cancer metabolism is the Warburg effect, which is characterized by the preferential conversion of glucose to pyruvate without engaging mitochondrial aerobic metabolism [19]. M1 macrophages rely heavily on glycolysis for fighting pathogens and tumor cells. The metabolic intermediates of aerobic glycolysis can be rerouted into oxidative pentose phosphate pathway (PPP), through which nicotinamide adenine dinucleotide phosphate (NADPH) is generated. NADPH-dependent reactive oxygen species (ROS) generation by NADPH oxidases (NOXs) is essential for the phagocytic and tumoricidal effects of M1 macrophages (Fig. 1) [20, 21]. The suppression of glycolysis and PPP attenuated LPS-induced inflammatory polarization of macrophage [22, 23], indicating the essential role of aerobic glycolysis and PPP in M1 macrophage differentiation. Glycolysis and PPP may promote the inflammatory response in macrophages by mediating NOX2 oxidase activity and IFN-β-dependent responses [24]. A recent study demonstrated that RNA-binding motif 4 (RBM4), an mRNA binding protein interacting with signal transducer and activator of transcription 1 (STAT1) mRNA, can suppress IFN-γ-induced M1 macrophage polarization by destabilizing STAT1 mRNA and downregulating glycolysis-related genes [25]. Nevertheless, the detailed mechanisms how aerobic glycolysis and PPP orchestrates the gene programs in M1 macrophages remain unclear.

Although M2 macrophage preferentially rely on fatty acid oxidation and mitochondrial metabolism, glycolysis is also required for supporting M2-like phenotype. Glucose uptake is increased over time in IL-4 polarized macrophages [26], and glycolysis inhibition by 2-Deoxy-d-glucose (2-DG) suppresses the M2 polarization [26–28]. In human melanoma-derived TAMs, accelerated aerobic glycolysis is required to support the M2-like polarization since glycolysis inhibition dampens the expression of M2 markers [29]. Nevertheless, there is evidence that glycolysis is not mandatory for the M2 polarization of macrophages if mitochondrial activity and oxidative phosphorylation (OXPHOS) remains intact [30]. The discrepancy of these studies may result from the differential effect of glucose deprivation and glycolysis inhibition by 2-DG. Although 2-DG is widely used as a glycolytic inhibitor, it is not specific and could affect OXPHOS differentially at different doses [30]. Genetic perturbation of glycolytic genes would be a superior approach to show the necessity of glycolysis in supporting functional M2 macrophage. Together, current knowledge favors the notion that glycolysis serves as a metabolic support for OXPHOS in the M2 polarization.

Although TAMs are believed to predominantly rely on OXPHOS and fatty acid oxidation (FAO) to metabolically support the pro-tumorigenic phenotype in a glucose-deficient TME, recent evidence from in vivo glucose tracing demonstrated the greatest glucose uptake capacity of myeloid cells in the TME and the preferential usage of glutamine by cancer cells, suggesting the nutrient partitioning by immune and cancerous cells [31]. Several lines of evidence also showed the upregulation of glycolytic genes in macrophages cultured in tumor-conditioning medium [32, 33]. Further, lactate production by glycolytic cancer cells can induce the upregulation of HIF-1α in TAMs to enhance the expression of glycolytic genes and M2-like state [34]. Therefore, glycolysis is an important metabolic process sustaining the functional phenotype of TAMs.

### The TCA cycle

The tricarboxylic acid (TCA) cycle is the key metabolic circuit of aerobic respiration in mitochondria. Following M1 polarization, macrophages show a highly glycolytic phenotype with reduced mitochondrial activity [35]. The diminished mitochondrial activity is associated with the truncated TCA cycling characterized by succinate accumulation and impaired metabolic flux through isocitrate dehydrogenase (IDH). It was found that inflammatory cytokines could upregulate aconitate decarboxylase 1 (ACOD1, also known as IRG1), an enzyme catalyzing itaconate (ITA) production from cis-aconitate of the TCA cycle during M1 macrophage polarization [36, 37]. ITA serves as a direct inhibitor of succinate dehydrogenase (SDH), leading to succinate accumulation during macrophage inflammatory activation [38]. IRG1-deficient macrophages without ITA synthesis showed increased cytokine production after being stimulated with LPS, suggesting an anti-inflammatory function of ITA. Since succinate accumulation stabilizes HIF-1α and increases the production of IL-1β [39], the truncated TCA cycle at succinate breakpoint can support the inflammatory polarization of macrophages, while ITA could fine tune the inflammatory responses. The impaired TCA cycle is also accompanied by an active aspartate-arginosuccinate shunt which relies on aspartate aminotransferase to produce L-arginine for nitric oxide (NO) synthesis [35]. Herein, the truncated TCA cycle supplies the intermediate for ROS production in M1 polarization.

In contrast, M2-polarized macrophages contain more mitochondria and show elevated oxygen consumption rate [40]. Unlike M1 macrophages which depend on glycolysis to fuel the TCA cycle, M2 macrophages tend to utilize glutamine to drive the TCA cycle [35]. Indeed, the M2 polarization remains unaffected in the deprivation of glucose if the mitochondrial activity and OXPHOS are preserved [30]. The increased number of functional mitochondria and the integrity of the TCA cycle in M2 macrophages allow M2 macrophages to exhibit significant plasticity and easily repolarize to the M1 state [41]. M1 macrophages with diminished OXPHOS and mitochondrial activity are resistant to M2 repolarization. Of note, different from L-arginine-dependent NO generation in M1 macrophages, M2 macrophages produce polyamines and L-proline from L-arginine to suppress inflammation [42]. Blocking NO production improves mitochondrial activity and facilitates the reprogramming from M1 to M2 state [41]. These observations collectively indicate that TCA cycle rewiring not only underpins the functional divergence of macrophages, but also has implication in the functional plasticity.

In macrophages isolated from tumor tissues, both the oxygen consumption rate (OCR) and extracellular acidification rate (ECAR) are higher than tumor cells and tumor-infiltrated T cells [31], indicating the continuous firing of both aerobic glycolysis and mitochondrial activity in TAMs. There is also evidence that a subpopulation of TAMs can use lactate to fuel the TCA cycle [43]. TCA cycle is interconnected with a variety of metabolic pathways to contribute to the pro-tumorigenic functions in TAMs, which can be rewired to repolarize the state of TAMs.

### Fatty acid metabolism

Elevated lipid synthesis is considered as a metabolic hallmark in carcinogenesis [44], and an important metabolic feature of M2-like macrophages in tumor tissues is the increased FAO. Fatty acids can be obtained directly from the external microenvironment or synthesized through intracellular lipogenesis. During M2 polarization, fatty acid uptake by the scavenger receptor CD36 and the lipolysis provide carbon source for FAO to fuel the TCA cycle and support the OXPHOS [45]. There is a concomitant upregulation of genes in fatty acid uptake, lipolysis and FAO upon M2 polarization. FAO supports the pro-tumorigenic potential of TAMs, as the inhibition of FAO suppresses tumorigenesis by promoting the anti-tumorigenic property of TAMs [46]. A recent work further demonstrated that IL-4 polarized and tumor-associated macrophages show increased activity of protein kinase RNA-like ER kinase (PERK), which is required to sustain FAO and mitochondrial activity by promoting serine biosynthesis. The depletion of PERK impairs the immunosuppressive phenotype of TAMs by dampening FAO and mitochondrial respiration [47].

However, if fatty acid biosynthesis and FAO are simultaneously induced, macrophages tend to polarize into anti-tumorigenic direction [31]. The application of Toll-like receptor 9 (TLR9) agonist shows anti-tumor effect by driving the metabolic reprogramming in macrophages. TLR9 signaling activation enables both FAO and the shunting of TCA cycle intermediates for lipogenesis, and carnitine palmitoyltransferase 1 (CPT-1, for fatty acid import to mitochondria) and adenosine triphosphate citrate lyase (ACL, for converting citrate to acetyl-CoA) coordinate the metabolic flow of FAO and lipogenesis [48]. The de novo biosynthesis of cholesterol is believed to improve the fluidity of cell membrane and enhance anti-tumor phagocytosis in TAMs. These results provide novel insights into how the coupling of lipid catabolism and anabolism impinges on macrophage function. It remains to be clarified whether the biosynthesis of lipids other than cholesterol also regulates the activity of macrophages.

### Glutamine metabolism

Glutamine, the most abundant circulating amino acid in blood, is closely associated with the metabolic needs in cancer cells, such as the supply of metabolites in the TCA cycle and the production of antioxidant glutathione [49]. Glutaminolysis-dependent pathway preferentially promotes M2 polarization. IL-4 stimulation leads to the increased uptake of glutamine in macrophages, which may depend on the upregulation of glutamine transporter [50]. Glutamine deprivation exerts profound effects on M2 polarization, including the reduced expression of M2 markers and attenuated TCA cycle, whereas M1 polarization seems unaffected [35]. In another independent study, glutamine removal also impairs the expression of M2-specific markers after IL-4 stimulation, while the expression of M1-specific markers after LPS activation shows upregulation when compared to the macrophages activated in glutamine-replete medium [51]. Thus, glutamine is essential for M2 polarization. Further investigation revealed the critical role of glutamine catabolic product α-ketoglutarate (α-KG) in supporting M2 polarization. Inhibiting glutaminase 1 (an enzyme for glutamine hydrolysis) impairs M2 phenotype after IL-4 polarization, and the supplementation of dimethyl-αKG (an analog of α-KG) is able to restore M2 phenotype. As an intermediate metabolite in the TCA cycle, α-KG fuels the TCA cycle to increase FAO and OXPHOS in M2 macrophages and drives the epigenetic reprogramming of M2 genes in a histone methylation-dependent fashion. In addition, M2 macrophages favor α-KG accumulation by dampening the enzymatic activity of α-KG dehydrogenase, and the increased ratio of α-KG/succinate suppresses the expression of inflammatory genes by inhibiting Nuclear factor-κB (NF-κB) [51]. Similarly, a recent study reported that IL-4 dependent activation of mitochondria-localized sirtuin-3 deacetylates glutamate dehydrogenase 1 (GLUD1), which enhances GLUD1 activity to accelerate glutaminolysis and α-KG production, leading to the alternative activation of M2 macrophages [52]. Notably, the TCA cycle breakpoints, including attenuated isocitrate to α-KG conversion [35] and the shunting of cis-aconitate to itaconate [38], help maintain a low level of α-KG in M1 macrophages. Therefore, α-KG serves as a metabolic hub of tailoring macrophage immune responses.

Since tumor cells are highly addicted to glutamine [31], how TAMs compete with tumor cells for glutamine is elusive. A recent work revealed that glutamine-addicted ovarian cancer cells secrete N-acetylaspartate metabolite which acts as a signaling molecule to upregulate glutamine synthetase (GS) in TAMs and support the M2-like state [53]. Macrophage-specific ablation of GS in tumor-bearing mice redirects TAMs toward an anti-tumorigenic M1-like state [54, 55]. Intriguingly, under glutamine-deprived condition macrophages tend to overexpress GS to replenish cellular level of glutamine [55, 56]. Therefore, glutamine overconsumption by tumor cells creates a glutamine-deficient TME where the induced GS expression might support the pro-tumorigenic state of TAMs.

## Signaling pathways involved in macrophage metabolic reprogramming

**Fig. 2:**
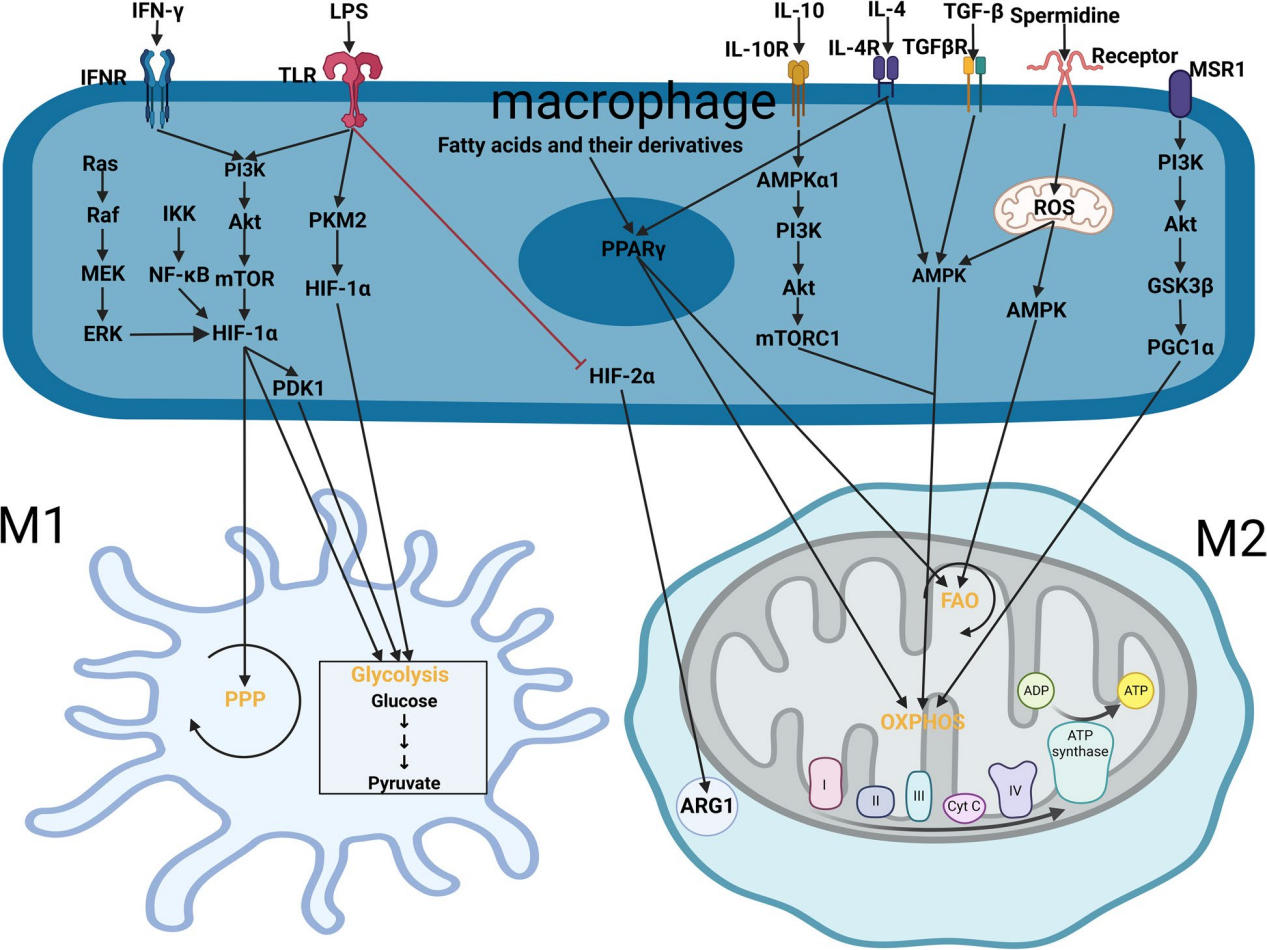
Signaling pathways implicated in macrophage metabolic reprogramming. M1 macrophages are classically polarized by IFN-γ and LPS, and the activation of PI3K-AKT-mTOR-HIF-1α signaling cascade sustains glycolysis and PPP. In contrast, the activation of PPARs and AMPK signaling underpins FAO and mitochondrial activity in IL-4/IL-10 polarized M2 macrophages. Abbreviations: AKT, protein B; AMPK, adenosine 5'-monophosphate–activated protein kinase; ARG1, arginase 1; ERK, extracellular regulated protein kinases; FAO, fatty acid oxidation; GSK3β, glycogen synthase kinase-3 beta; HIF, hypoxia inducible factor; IFN-γ, interferon γ; IFNR, interferon receptor; IKK, inhibitor of kappa B kinase; IL, interleukin; IL-10R, interleukin-10 receptor; IL-4R, IL-4 receptor; LPS; lipopolysaccharide; MEK, mitogen-activated protein kinase kinase; MSR1, macrophage scavenger receptor 1; mTOR, mammalian target of rapamycin; mTORC1, mTOR complex 1; NF-κB, Nuclear factor-κB; OXPHOS, oxidative phosphorylation; PDK1, pyruvate dehydrogenase kinase isozyme 1; PGC1α, proliferator-activated receptor gamma coactivator 1 α; PI3K, phosphatidyl inositol 3-kinase; PKM2, pyruvate kinase 2; PPARγ, peroxisome proliferator activation receptor gamma; PPP, pentose phosphate pathway; Raf, rapidly accelerated fibrosarcoma; Ras, rat sarcoma; ROS, reactive oxygen species; TCA, tricarboxylic acid; TGF-β, trans-forming growth factor-β; TGFβR, TGF-β receptor; TLR, Toll-like receptor (Created with BioRender.com)

Macrophages have been conventionally classified into two phenotypes based on their activation status and function. M1 macrophages are classically differentiated and activated by interferon gamma (IFN-γ, IFNG) and lipopolysaccharide (LPS), while M2 macrophages are alternatively activated by T helper cell 2 (Th 2) cytokines, including interleukin-4 (IL-4) and Interleukin-13 (IL-13). The functional polarization is accompanied by the metabolic rewiring, manifesting as the preferential glycolysis in M1 macrophages and the domination of OXPHOS and FAO in M2 macrophages. In this section, we summarize the signaling pathways implicated in macrophage metabolic reprogramming, mainly focusing on phosphatidyl inositol 3-kinase(PI3K)/protein kinase B (AKT) pathway, hypoxia inducible factor (HIF), adenosine 5'-monophosphate–activated protein kinase (AMPK) and peroxisome proliferator activation receptors (PPARs) (Fig. 2).

### PI3K/AKT/mTOR Pathway

Since Bellacosae et al. Characterized AKT as an oncogene 32 years ago [57] and Franke et al. identified PI3K as its upstream regulator [58], the roles of PI3K/AKT in various cell types and cellular processes have been expensively studied. The regulation of PI3K/AKT pathway in macrophages is not only restricted to cell survival, migration and proliferation, this pathway also engages in the metabolic responses to inflammatory signals [59]. Signals from Toll-like receptors (TLRs), cytokines, Fc receptors (FCR) and other pathogen recognition receptors activate PI3K [59–61]. The activated kinase activity of PI3K converts phosphatidylinositol 4,5-bisphosphate (PIP2) to phosphatidylinositol 3,4,5-triphosphate (PIP3) at the plasma membrane. PIP3 serves as the membrane anchor for AKT which is then activated by pyruvate dehydrogenase kinase isozyme 1 (PDK1) and mammalian target of rapamycin complex 2 (mTORC2) [61]. LPS and IFN-γ stimulation causes the metabolic shift towards glycolysis and PPP in M1 polarization in vitro, a process dependent on PI3K/AKT signaling, and glycolysis inhibition suppresses the inflammatory polarization in macrophages [62]. Silencing AKT attenuates glycolytic shift and macrophage activation during M1-like polarization [63], suggesting the pivotal role of AKT-dependent metabolic pathway in classical M1 polarization.

mTOR is a serine/threonine protein kinase and the downstream effector of PI3K and AKT signal transduction. mTOR interacts with other protein adaptors to form two different complexes, mTORC1 (with Raptor) and mTORC2 (with Rictor) [64]. mTORC1 activation is known to promote the expression of metabolic genes in glycolysis and PPP [65]. However, the role of mTORC1 in macrophage polarization is controversial. As shown by pharmaceutical inhibition, mTORC1-dependent glycolysis is indispensable for the inflammatory polarization of macrophage induced by LPS [66], In addition, PI3K/AKT/mTOR-mediated aerobic glycolysis is essential for the persistent inflammatory phenotype of M1-like macrophages in the mouse model of trained immunity [67]. On the other hand, IL-4 signaling also activates AKT-mTORC1 pathway to phosphorylate and activate ATP-citrate lyase (ACLY, a key enzyme in converting citrate to acetyl-CoA), culminating in histone acetylation at M2 gene loci. Nevertheless, only a subset of M2 genes is regulated by this manner, indicating that AKT-mTORC1-Acly axis fine tunes metabolic state to control M2 activation [26]. A recent study employing genetic deletion of mTORC1 in mouse macrophage revealed an augmented M1 macrophage phenotype despite the impaired glycolysis, which is linked to the epigenetic activation of M1 genes through enhanced histone acetylation [68]. The discrepancy of these studies may come from the difference between the cell and animal models, and between the genetic ablation and pharmaceutical inhibition. In contrast, mTORC2 has a more definite role in dictating M2-like polarization in macrophage. IL-4 polarized macrophages show enhanced FAO and OXPHOS, coupled with augmented glycolysis. The activation of mTORC2 is required for the increased glucose uptake in M2 macrophages [28]. Macrophage colony stimulating factor (M-CSF) seems to act as an upstream ligand to activate mTORC2 through PI3K/AKT pathway. Genetic deletion of Rictor (mTORC2 adaptor) suppresses the production of M2-like macrophages in the mouse model, while the generation of M1-like pro-inflammatory macrophages remains intact [28, 69]. Therefore, mice with macrophage-specific Rictor deletion show exaggerated sensitivity to LPS-induced sepsis [70], impaired clearance of parasitic nematode [69], enhanced inflammatory cytokine production as well as the suppression of tumor growth [28, 71]. The divergent roles of mTORC1 and mTORC2 in macrophage polarization provide plausible target for manipulating macrophage activation. In addition to mTOR-dependent metabolic regulation, Shu-Jie Zhao et al. reported that proliferator-activated receptor gamma coactivator 1-alpha (PGC1α) is a target gene of PI3K/AKT/GSK3β/β-catenin pathway activated by macrophage scavenger receptor 1 (MSR1), which promotes M2-like differentiation by enhancing mitochondrial OXPHOS [72]. Nevertheless, the mechanism by which MSR1 activates PI3K/AKT pathway remains to be clarified.

There is evidence that PI3K/AKT/mTOR signaling axis mediates the infiltration and activity of TAMs. In melanoma, elevated level of TGF-β was reported to activate PI3K/AKT signaling and promote the tumor infiltration of immune-suppressive monocytes by upregulating monocyte chemoattractant protein-1 (MCP-1) expression and IL-10 [73]. Tamoxifen-resistant breast cancer cells activate mTORC1 signaling by altering amino acid metabolism to favor M2 macrophage polarization [74]. DNA Damage Inducible Transcript 4 (REDD1, DDIT4), a negative regulator of mTORC1, is upregulated in TAMs to suppress glycolysis. REDD1-deficient TAMs exhibited highly glycolytic features and increased glucose uptake in an mTOR-dependent manner, which impinges on neovascularization and tumor metastasis; however, the tumor growth remains unaffected [14].

### HIF

HIF is a heteroprotein dimer consisting of α subunit and β subunit. The expression level of α subunit is oxygen-dependent, while the β subunit is constitutively expressed [75]. Under normoxia, HIF-1α is hydroxylated by prolyl hydroxylase (PHD) and degraded by the ubiquitin-dependent process. In the hyopixc environment, HIF-1α is stabilized to promote glycolysis by transcriptionally upregulating glucose transporter as well as glycolytic genes (such as hexokinase, phosphofructokinase, and pyruvate kinase) [76]. In addition, HIF-1α is a downstream signaling molecule of multiple signaling pathways (including PI3K/AKT/mTOR, Ras/Raf/MEK/ERK(MAPK) and IKK/NF-κB), which are trigger by different inflammatory cytokines [75, 77]. LPS stimulation stabilizes HIF-1α during M1 macrophage differentiation, which is accompanied by a metabolic shift toward glycolysis and PPP [39, 78]. Wang et al. showed that mitochondrial activity was diminished in HIF-1α overexpressing macrophages, as evidenced by the reduced OCR. The elevated ECAR indicates the boosted aerobic glycolytic metabolism in macrophages with HIF-1α overexpression, which sustains the M1 polarization. The team also found increased levels of metabolic intermediates in glycolysis and PPP, as well as the upregulation of glycolytic genes [78]. In another study, under mild hypoxic condition during macrophage migration to the inflammatory sites, HIF-1α stabilization was found to promote glycolysis by increasing the expression of PDK1 which prevents pyruvate from entering the TCA cycle [79]. Inhibiting glycolysis undermines macrophage migration and attenuates systemic inflammation. Enhanced glycolysis could in turn promote the stabilization or enhance the activity of HIF-1α. Accelerated glycolysis results in the accumulation of succinate in TCA cycle, and elevated succinate levels inhibit PHD due to the competitive binding to its active site [80]. Furthermore, pyruvate kinase 2 (PKM2), a glycolytic enzyme upregulated by LPS induction, can translocate into the nucleus and interacts with HIF-1α to promote the transcription of target genes. Inhibiting PKM2-HIF-1α interaction impaired glycolysis and diminished the production of pro-inflammatory cytokine IL-1β after LPS stimulation [81]. Therefore, HIF-1α-depndent glycolysis forms a positive-feedback loop to stabilize the metabolic reprogramming in M1 polarization.

The role of another HIF α subunit (HIF-2α) in the metabolic reprogramming of macrophage is obscure. There is evidence that HIF-1α and HIF-2α are differentially activated in M1 and M2 polarization. LPS and IFN-γ stabilize HIF-1α and suppress HIF-2α gene expression, while IL-4 and IL-13 increase HIF-2α protein level [82]. HIF-2α can induce arginase 1 (ARG1) gene expression in M2 macrophages, the enzyme competing with inducible nitric oxide synthase (iNOS) for L-arginine metabolism and thereby limiting NO production [83]. Whether HIF-2α competes with HIF-1α or orchestrates other metabolic pathways in macrophage polarization needs further clarification.

It is intuitive to speculate that the hypoxic TME causes the stabilization of HIFs to impinge on the metabolic reprogramming of TAMs. Indeed, clinical evidence suggests the preferential upregulation of HIF-1α and HIF-2α in TAMs [84, 85]. Genetic ablation of HIF-1α in TAMs reinforces the M2 features and attenuates the cytotoxic effect towards tumor cells [86]. A puzzle remaining to be resolved is how the activation of HIFs orchestrates the metabolic programs in TAMs to support the pro-tumorigenic activity.

### AMPK

AMPK is a conserved serine/threonine kinase consisting of three distinct subunits of catalytic α (α1, α2), regulatory β (β1, β2) and γ (γ1, γ2, γ3) [87]. Apart from acting as an energy sensor for ADP/ATP ratio, AMPK also serves as the kinase of several signaling cascades activated by IL-10, IL-4, TGF-β and oxidative stress, which promotes OXPHOS in M2 polarization [88–91]. Anti-inflammatory cytokines (IL-10 and TGF-β) induces rapid phosphorylation of AMPK in macrophages, whereas LPS stimulation results in AMPK inactivation [92]. Silencing AMPK in macrophages augments LPS-induced inflammatory responses, while constitutive activation of AMPK shows the opposite effect, suggesting the anti-inflammatory effect of AMPK signaling. Recently, R. Liu et al. reported that spermidine, a natural polyamine, is able to activate AMPK to support anti-inflammatory polarization in macrophages. Spermidine treatment enhanced the production of mitochondrial ROS to activate AMPK, which leads to the upregulation of the components in OXPHOS and FAO, as well as the total mass of mitochondria. Blocking AMPK activity attenuated the effect of spermidine on mitochondrial activity and the anti-inflammatory differentiation in macrophages [89]. In addition, spermidine suppressed LPS-mediated pro-inflammatory responses in an AMPK-dependent manner, which is consistent with the previous observation that AMPK functions as a negative regulator of LPS-induced inflammatory responses in macrophages [92].

IL-10 is a signal molecule upstream of PI3K/AKT/mTORC1 pathway in macrophages, and IL-10 promotes AMPKα1 phosphorylation to activate this pathway. The activation of PI3K/AKT/mTORC1 pathway by IL-10 promotes OXPHOS to suppress inflammatory phenotype in macrophages [90], while LPS activates PI3K pathway to promote glycolysis and inflammatory responses [61], suggesting that there are additional players mediating differential metabolic rewiring induced by different signaling molecules. The application of PI3K inhibitor does not affect IL-10-depnedent AMPKα1 activation, and AMPKα1 seems to function in parallel to PI3K pathway to orchestrate the anti-inflammatory phenotype by phosphorylating STAT3 [91], However, a recent study in bacterial infection model demonstrated that AMPK activated by vascular endothelial growth factor C (VEGFC) signaling promotes glycolysis and inflammasome activation in macrophages to facilitate the clearance of bacteria [93]. Thus, AMPK is a critical mediator of metabolic reprogramming in macrophage polarization, and its role in different pathophysiological conditions warrants further investigation.

### PPARs

PPARs are nuclear hormone receptors usually activated by fatty acids and their derivatives, which are engaged in the metabolic reprogramming of macrophages. According to different structures, PPARs can be divided into three subtypes: α, β/δ and γ [94]. PPARγ is the main subtype regulating fatty acid metabolism of macrophages. An early study by Szanto et al. demonstrated that pro-inflammatory factors suppress PPARγ expression in both mouse and human macrophages, while IL-4 enhances PPARγ expression and the ligand-induced transcriptional activity [95]. It is well-documented that the activation of PPARγ by different signaling supports the anti-inflammatory polarization in macrophages by enhancing OXPHOS and FAO. S. Kangetal et al. reported that the inhibition of mTOR or the deletion of Semaphorin 6D impairs PPARγ expression, dampening fatty acid metabolism and blocking the polarization of anti-inflammatory macrophages [96]. The transcriptional activity of PPARγ is indispensable for the activation of metabolic genes, such as ARG1 and genes involved in fatty acid β-oxidation and mitochondrial biogenesis [96]. In the TME, PPARγ-dependent enhancement of FAO mediates the anti-inflammatory and pro-tumorigenic properties of TAMs [97]. In hepatocellular carcinoma, the functional deficiency of receptor-interacting protein kinase 3 (RIPK3) inhibits caspase-1 dependent PPAR cleavage, and the accumulation of PPAR augments the FAO and M2-like polarization of TAMs. RIPK3 upregulation or FAO blockade reversed the immunosuppressive activity of TAMs and dampened HCC tumorigenesis [98]. There is also evidence that M-CSF secreted from tumor cells upregulates PPARβ/δ expression in myeloid cells to promote IL-10 expression and induce the polarization of M2-like macrophages [99]. These studies pinpoint the potential of targeting PPAR signaling to reverse the immunosuppressive phenotype of TAMs.

## Extrinsic metabolites dictating macrophage polarization in the TME

**Fig. 3:**
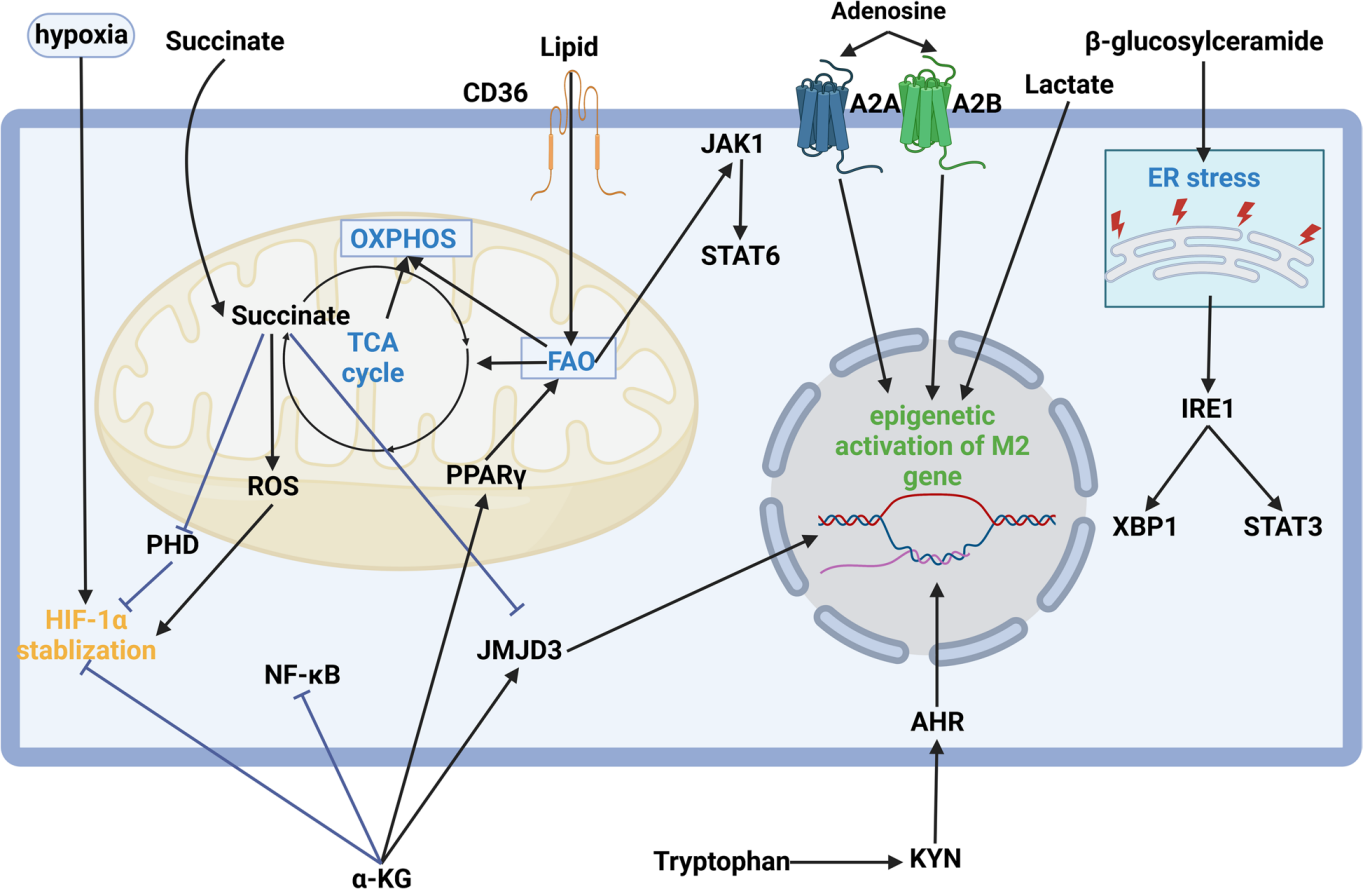
Functional polarization of tumor-associated macrophages (TAMs) is influenced by multiple metabolites present in the TME. Extracellular metabolites not only serve as energy sources but also act as environmental cues to regulate the immune phenotype of TAMs. Lipids, succinate, α-Ketoglutarate, amino acids, adenosine and lactate are all implicated in fine-tuning TAM function. Abbreviations: α-KG, α-ketoglutarate; ERS, endoplasmic reticulum stress; FAO, fatty acid oxidation; HIF-1α, hypoxia inducible factor-1α; IRE1, inositol-requiring enzyme 1; ITA, itaconate; JAK, Janus Kinase 1; OXPHOS, oxidative phosphorylation; PHD, prolyl hydroxylase; PPARγ, peroxisome proliferator activation receptor gamma; ROS, reactive oxygen species; SDH, succinate dehydrogenase; STAT3, signal transducer and activator of transcription; TCA, tricarboxylic acid; XBP1, x-box binding protein 1 (Created with BioRender.com)

In the TME, the crosstalk of different cellular components shapes the metabolic milieu, which has widespread implications in tumor progression and the anti-tumor immunity. Extracellular metabolites in the TME not only serve as energy sources but also act as signaling cues to regulate the immune phenotype of TAMs. In this section, we focus on TME metabolites that influence macrophage metabolic reprogramming, including lipids, succinate, α-Ketoglutarate, amino acids, adenosine and lactate (Fig. 3 and Table 1).

**Table 1:** The impacts of extrinsic metabolites on the metabolic reprogramming in TAMs

Metabolite	Signaling pathway	Metabolic pattern	Polarization	Ref
Lipids	**JAK1/STAT6; mTOR; IRE1-XBP1/STAT3**	**FAO; Mitochondrial OXPHOS; ER lipid reshuffling and stress**	**Pro-tumorigenic polarization**	[100–102]
Succinate	**SUCNR1/PI3K/HIF-1α**	**Mitochondrial TCA cycle and glycolysis**	**Pro-tumorigenic polarization**	[103]
α-KG	**JMJD3; NF-κB; PPARγ**	**Mitochondrial respiration and FAO**	**Pro-tumorigenic polarization**	[51, 104]
Glutamine	**_**	**Glutaminolysis and α-KG production**	**Pro-tumorigenic polarization**	[35, 55]
Tryptophan	**AHR**	**Tryptophan catabolism and KYN production**	**Pro-tumorigenic polarization**	[105]
Adenosine	**Adenosine receptors (A2A and A2B)**	**_**	**Pro-tumorigenic polarization**	[106, 107]
Lactate	**HIF-1α**	**Lactate metabolism in TCA cycle and histone lactylation**	**Pro-tumorigenic polarization**	[34, 108, 109]

### Lipids

Tumor tissues are enriched in lipids due to the de novo lipogenesis of cancer cells, and the lipid supply of cancer-associated fibroblasts and adipocytes [100, 101, 110, 111]. TAMs are overloaded with lipids due to increased lipid uptake by the scavenger receptor CD36, and TAMs preferentially rely on FAO for energy [100, 101, 111]. Both lipid accumulation and high CD36 expression are correlated with the immunosuppressive function of TAMs and unfavorable tumor progression. Targeting intratumoral lipid droplet formation or genetic deletion of CD36 curbs the pro-tumoral function of TAMs and suppresses tumor progression [100, 101, 111]. High levels of FAO support TAM generation by promoting mitochondrial OXPHOS and inducing JAK1 (Janus Kinase 1)/STAT6 activation [100]. Fatty acids such as oleate induce pro-tumoral polarization of TAMs by augmenting mTOR-dependent mitochondrial respiration [101]. Another study showed that ovarian cancer cells promote plasma membrane cholesterol efflux from TAMs, and the subsequent loss of cholesterol-rich membrane lipid rafts activates IL-4 signaling while suppressing IFNγ-induced genes. IL-4 signaling and cholesterol efflux pathways contribute to the immune suppression of TAMs and tumor progression in vivo [112]. In our recent work, it was found that β-glucosylceramide produced by tumor cells drives the reorganization of lipid components on endoplasmic reticulum (ER) membrane, resulting in IRE1 (inositol-requiring enzyme 1)-dependent ER stress responses. Targeting IRE1-XBP1 (x-box binding protein 1) and IRE1-STAT3 signaling or ameliorating ER stress through genetic perturbation can disrupt the pro-tumoral activity and survival of TAMs [102]. These findings pinpoint targeting ER lipid composition and responses as potential strategy to sustain anti-tumor immunity.

### Succinate

Succinate is an intermediate metabolite of the TCA cycle and has been considered as a pro-inflammatory metabolite. It stabilizes HIF-1α by inhibiting PHD activity and promoting ROS production [38, 39]. SDH is the catalyst for succinic acid production in the TCA cycle. In macrophage inflammatory polarization, SDH inhibition by ITA leads to succinate accumulation and HIF-1α dependent metabolic changes [38, 113]. The frequent mutations of the gene encoding SDH in cancers result in the accumulation of succinate in the TME [114]. Although macrophage-intrinsic succinate accumulation was widely reported as an inflammatory modulator in M1-polarized macrophages, a recent study showed the pro-tumoral effect of extracellular succinte on TAMs [103]. Cancer cells secrete succinate into the TME to promote the migration and invasion by activating succinate receptor (SUCNR1) signaling. In the meanwhile, the activation of PI3K/AKT/HIF-1α signaling underpins succinate-induced TAM polarization to support cancer cell migration. Although the authors could not exclude the influence of other tumor-derived small molecules on TAMs, they provided evidence that increased serum succinate concentration may serve as a biomarker for lung cancer [103]. There is also evidence that succinate fuels mitochondrial oxidation via SDH and the concomitant mitochondrial membrane potential elevation drives mitochondrial ROS production [115], which may serve as a key mechanism for maintaining the pro-inflammatory state of macrophages [116, 117]. Hence, macrophage-intrinsic succinate accumulation due to truncated TCA cycle and the extracellular succinate overload may differentially regulate TAM function.

### α-Ketoglutarate

α-KG is a decomposition product of glutaminolysis and an intermediate product of the TCA cycle. α-KG promotes M2 polarization through Jumonji-C-domain-containing histone demethylase 3 (JMJD3)-dependent epigenetic reprogramming, and impairs the pro-inflammatory response of M1 macrophages by inhibiting the NF-κB pathway [51]. After M2 polarization, the expression of FAO rate-limiting enzyme carnitine palmitoyltransferase 1A (Cpt1a) increases with enhanced fatty acid uptake in a α-KG-dependent manner. Similarly, α-KG supplementation suppressed M1 signature gene expression and dampened the activation of mTORC1/p70 ribosomal protein S6 kinase (p70S6K/S6K1) signaling in M1-polarized MH-S cells (a murine alveolar macrophage cell line) [104]. Further evidence showed that α-KG promoted FAO and M2 polarization by enhancing the nuclear translocation of PPARγ and increasing the expression of fatty acid metabolism genes. α-KG alone is able to offset the HIF-1α activation induced by succinate or hypoxia [118, 119], and α-KG is a co-stimulator of JMJD3 while succinate is an inhibitor [51]. Thus, the ratio of α-KG/succinate could be a determinant for the polarization state of macrophages. Indeed, an increase of α-KG/succinate ratio favors M2 polarization, while a decrease in the ratio strengthens the M1 phenotype [51]. As glutaminolysis has been recognized as a hallmark of cancer metabolism [120], it is speculated that α-KG derived from cancer metabolism could act as a TME metabolite to modulate TAM polarization. The ratio of α-KG/succinate in the TME and its impact on TAM function remain to be elucidated.

### Amino acids

A variety of amino acids are involved in the regulation of macrophage polarization and activation. Glycine was previously reported to suppress LPS-induced NO production and macrophage activation [121], and glycine regulates macrophage polarization via different signaling pathways and microRNAs [122]. Another one-carbon amino acid serine is required for macrophage IL-1β production [123]. Serine deprivation blunts macrophage IL-1β expression level by dampening mTOR signaling [124]. However, a recent work showed that the depletion of exogenous serine and glycine augmented M1 polarization but attenuated IL-4-polarized macrophages. Furthermore, macrophage-specific serine restriction was able to reprogram TAMs into the M1 phenotype and retard tumor growth [125].

Cancer cells are highly addicted to glutaminolysis for energy production [31, 120]. Glutamine deprivation could reduce M2 polarization and the production of the chemokine C–C motif chemokine ligand 22 (CCL22) [35]. This is in agreement with the M2-polarizing effect of α-KG as the decomposition product of glutamine [51]. In contrast, LPS-stimulated M1 differentiation does not require glutamine [35], and α-KG supplementation suppressed M1 activation in a murine alveolar macrophage cell line [104]. Glutamine is synthesized by GS using ammonia and glutamate. In the TME, GS enzyme activity is highly correlated with macrophage M2 polarization [126]. GS activity inhibition leads to phenotypic transformation of M2-polarizing macrophages towards to M1, which is coupled with elevated levels of succinate. Meanwhile, macrophage-specific deletion of GS in tumor-bearing mice promotes anti-tumor immunity and suppresses metastasis [55].

L-arginine is also a mediator of macrophage polarization and can be derived from glutamine through citrulline intermediates. Macrophages maintain their ability to secrete arginine through high concentrations of glutamine [127]. The enhanced catabolism of arginine by ARG1 pathway supports M2 polarization, while the increased NO production from arginine by iNOS promotes M1 polarization. It is worth noting that these two metabolic pathways mutually inhibit each other [128]. Although it has been shown that ARG1 is highly expressed in M2-like TAMs [129], little is known about how arginine availability affects macrophage polarization in the TME.

Tryptophan is another important amino acid that regulates adaptive immunity in the TME. In TAMs and intratumoral regulatory T cell (Tregs), the tryptophan metabolizing enzymes indoleamine-2,3-dioxygenase (IDO) and tryptophan 2,3-dioxygenase (TDO) break down tryptophan into kynurenine (KYN), a metabolite binding to aryl hydrocarbon receptor (AHR) to orchestrate immunosuppression in multiple immune components of the TME [130]. Recent evidence revealed that in IDH-mutant glioma, tryptophan catabolism along the kynurenine pathway drives the immunosuppressive function of TAMs, which can be reversed by pharmacological inhibition of tryptophan metabolism and AHR [105]. Along similar lines, TAMs from pancreatic ductal adenocarcinoma (PDAC) display high AHR activity and AHR deletion in macrophages promotes an inflammatory state. Intriguingly, macrophage-intrinsic tryptophan metabolism seems dispensable for the immunosuppressive activity of TAMs. But dietary restriction of tryptophan reduces AHR activity in TAMs and promotes anti-tumor immunity in PDAC mouse model [131]. These observations suggest that tryptophan availability is linked with the polarization state of TAMs, and tryptophan metabolites impose immunosuppression by activating AHR in the TME.

### Adenosine

Adenosine is a metabolite released from various cell types and is present at high levels in the TME [132]. Ectonucleoside triphosphate diphosphohydrolase 1 (CD39) and ecto-5ʹ-nucleotidase (CD73) expressed at the cell surface of macrophages and cancer cells in the TME are responsible for the adenosine generation [133]. Adenosine exerts a range of immunomodulatory effects on macrophages by engaging adenosine receptors (A1, A2A, A2B and A3). There is abundant evidence that adenosine activates A2A and A2B receptors to mediate M2-like macrophage polarization [106, 107, 134]. Knockout of the A2A receptor in myeloid cells dampens IL-10 production in TAMs and augments anti-tumor immunity in melanoma model, highlighting the critical role of adenosine signaling in orchestrating myelosuppressive effects in the TME [135]. Blocking CD39 or CD73 activity can attenuate TAM inhibition on T cell proliferation [136]. Of note, tumor-derived adenosine in the TME sustains macrophage proliferation by activating PI3K/Akt pathway [122], which may also induce metabolic changes in TAMs. Thus, adenosine can simultaneously regulate the proliferation and polarization state of TAMs.

### Lactate

Lactate generated by the aerobic glycolysis of tumor cells is a favorable factor for cancer progression [137]. Lactate accumulation contributes to immunosuppression in the TME by dampening tumoricidal effects of tumor-infiltrating immune cells [138]. Tumor-derived lactate drives macrophage M2 polarization and induces VEGF expression in a HIF-1α-dependent manner [34]. In addition, Zhang et al. found that lactic acid metabolism is linked with histone modification to fine tune macrophage polarization. M1 macrophages undergo lactate anabolism using glycolysis to produce lactyl-CoA, while in the late stage of M1 polarization the increased histone lactylation is associated with M2 signature gene activation [108]. Exogenous lactate can also directly activate M2-like gene expression through histone lactylation. Hence, lactate-dependent epigenetic mechanism serves as a negative feedback to prevent the over-activation of inflammatory macrophages. The high content of lactic acids in the TME may also favor M2-like polarization in TAMs. Another study demonstrated that M2 macrophages actively metabolize lactate in the TCA cycle to support histone acetylation-mediated M2 gene expression [109]. IL-4-induced M2 polarization activates mitochondrial lactate metabolism in the TCA cycle. Citrate is then shunted away from the TCA cycle for acetyl-CoA production by ACLY, with subsequent histone acetylation at M2 gene promoters. Therefore, lactate can be utilized in mitochondrial metabolism of macrophages and ACLY-dependent acetyl-CoA production from lactate induces M2 genes via histone acetylation. Importantly, ACLY deficiency impairs the immunosuppressive activity in TAMs, suggesting the critical role of lactate-dependent metabolic circuit in TAM polarization [109]. In addition, lactate concentration gradient in the TME can transmit spatial information to instruct macrophage polarization [139], which may regulate the functional diversity of TAM subpopulations.

## Metabolic reprogramming of TAMs as an anti-cancer therapy

Using TAMs or their functional mediators as direct targets, various therapeutic strategies have been developed to overcome immunosuppression in the TME, including depleting TAMs, blocking monocyte/macrophage recruitment, and reprogramming TAMs into pro-inflammatory M1-like macrophages [140, 141]. Since metabolic alterations are the main drivers of macrophage suppression in the TME [142], repolarization of TAMs via metabolic reprogramming presents the opportunity to activate tumoricidal immunity.

Glutamine synthase (GS) is a critical enzyme driving M2-like macrophage differentiation by elevating glutamine level. It has been showed that inhibiting GS by methionine sulfoximine (MSO) skews M2 macrophages towards an M1-like phenotype in IL10-treated macrophages [55]. GS inhibition induces a metabolic rewiring involving glucose shunting into the TCA cycle and succinate accumulation. The elevated succinate level favors pro-inflammatory polarization of macrophages through the inhibition of anti-inflammatory gene expression and stabilization of HIF-1α. In the Lewis lung carcinoma model, macrophage-specific ablation of GS facilitates M1-like reprogramming in TAMs and leads to intratumoral cytotoxic T cell (CTL) accumulation [55]. Our study implied that the production of αKG via glutamine catabolism is important for the JMJD3-dependent epigenetic activation of M2 genes. A low ratio of α-KG/succinate strengthens M1 macrophage activation, whereas a high ratio favors M2 macrophage function [51]. Therefore, modulating the ratio of αKG/succinate can be exploited to fine tune the immune responses of TAMs.

Lactate impinges on macrophage metabolism via Gpr132, a macrophage sensor of the rising lactate to promote M2-like phenotype in TAMs [143]. Pharmacological inhibition or genetic deletion of Gpr132 could attenuate M2-like phenotype in TAMs and impair the tumor formation of breast cancer cells. Along this line, reducing lactate level in the TME by deleting lactate dehydrogenase A (LDHA) or through the administration of 2-deoxyglycose potentiates anti-tumor immunity by decreasing M2-like macrophage polarization [144, 145].

Additional strategy to reprogram TAMs includes the modulation of arginine catabolism. Inhibition of ARG1 by CB-1158 was able to shift the TME towards a pro-inflammatory environment by blunting myeloid cell-mediated immune suppression [146]. Although iNOS-derived NO has tumoricidal function, the selective inhibitor of iNOS was reported to enhance M1 macrophage polarization; while NO donor inhibited M1 macrophage polarization [147]. NO derived from iNOS mediates the nitration of interferon regulatory factor 5 (IRF5) protein and suppresses IRF5-induced M1 signature genes.

**Fig. 4:**
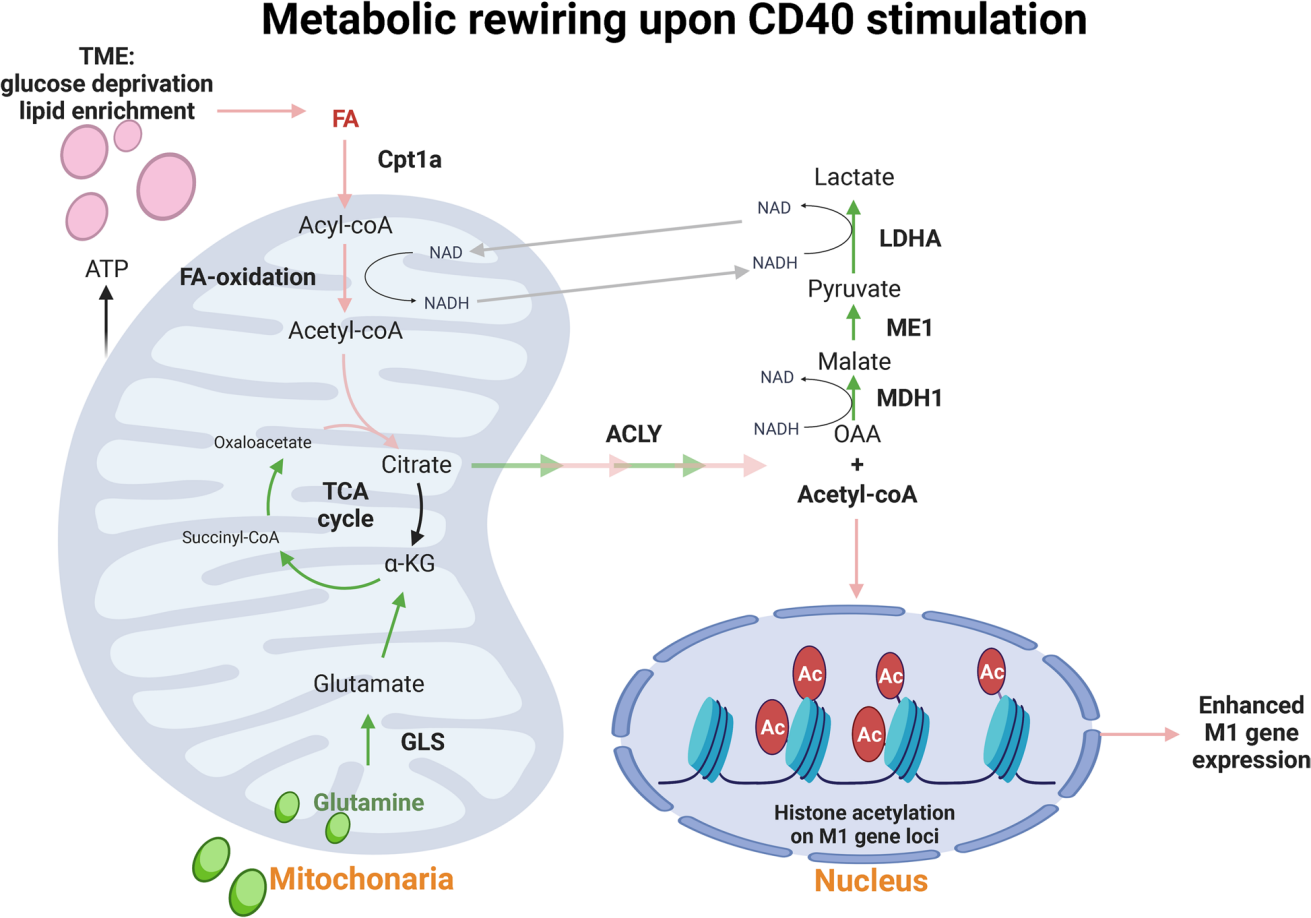
Schematics of CD40 signaling-induced metabolic rewiring to support anti-tumorigenic functions of macrophages. CD40 activation engages glucose-independent metabolic pathways to induce epigenetic activation of pro-inflammatory M1 genes. CD40 signaling triggers FAO and glutamine metabolism to drive the TCA cycle. The citrate is shunted towards ACLY-dependent acetyl-CoA production to promote histone acetylation and M1 gene activation. Glutamine usage reinforces FAO-dependent anti-tumorigenic functions by maintaining the NAD + /NADH ratio via lactate pathway. Abbreviations: ACLY, ATP-citrate lyase; ATP, Adenosine triphosphate; Cpt1a, carnitine palmitoyltransferase 1A; FA, fatty acid; GLS, glutaminase; LDHA, lactate dehydrogenase A; ME1, malic enzyme 1; MDH1, malate dehydrogenase 1; OAA, Oxaloacetic acid; TCA: tricarboxylic acid (Created with BioRender.com)

In our recent work, we showed that CD40 signaling activation by monoclonal antibody rewires metabolic circuits to enhance the anti-tumorigenic polarization of TAMs and boost anti-tumor response (Fig. 4) [148]. In contrast to LPS-polarized M1 macrophages which are highly dependent on glucose, CD40 signaling-mediated pro-inflammatory polarization is glucose-independent. Instead, CD40 signaling promotes both FAO and glutamine metabolism to instruct epigenetic reprogramming for pro-inflammatory/anti-tumorigenic polarization in macrophages. Mechanistically, CD40 activation augments histone acetylation on the promoters and enhancers of pro-inflammatory signature genes using acetyl-CoA produced by FAO. In parallel, CD40 signaling triggers glutaminolysis-dependent production of lactate in the absence of glucose. Intriguingly, glutamine-derived lactate production is critical to sustain FAO by fine-tuning nicotinamide adenine dinucleotide (NAD) / nicotinamide adenine ainucleotide hydrogen (NADH) balance. We also provided evidence that metabolic interventions by depleting LDHA and GLS (a key enzyme of glutaminolysis), which are two metabolic targets under investigations in clinical trials for cancer therapy, abrogated the anti-tumor response of TAMs induced by agonistic anti-CD40 antibody [148]. Thus, CD40 signaling harnesses metabolic processes (FAO and glutaminolysis) which are believed to support M2 polarization to orchestrate pro-inflammatory polarization in macrophages. Of note, these findings highlight that properly pre-conditioned metabolic milieus in the TME may potentiate the anti-tumor effect of agonistic anti-CD40 antibody.

Preclinical evaluation of metabolic reprogramming of TAMs has shown promising effects with the drugs targeting arginine and tryptophan metabolism [105, 131, 149, 150], respiratory complex I inhibitor metformin [151, 152], inhibitors of extracellular adenosine and lactate production [153, 154], and FAO inhibitor [101, 155]. Although most strategies for targeting TAMs are still in the preclinical stage, a number of therapeutic approaches (e.g., CD40 agonists, HDAC inhibitors, PI3Kγ inhibitors) are under evaluation in clinical trials in conjugation with immune checkpoint therapy [156]. Macrophage-targeting approaches also synergize with chemotherapeutics to reduce tumor burden and improve the survival in tumor-bearing mice [9]. Since there is accumulating evidence that dietary pattern generates a profound effect on TAMs and anti-cancer immunity [157, 158], we surmise that evidence-based dietary specification for cancer patients could be beneficial to maximize the effect of immunotherapy.

## Conclusion

It has become a consensus that metabolic alterations are integral components accompanying macrophage polarization. After recruitment to the TME, TAMs inevitably rewire the metabolic network to support their survival and differentiation. This process can be influenced by TME-derived metabolites such as adenosine, lactate and lipids. On the other hand, TAM-intrinsic metabolic changes and the accumulation of certain metabolites, such as succinate and α-KG, may also function to fine-tune or reinforce the functional differentiation. The plasticity of metabolic profile during the functional polarization in TAMs constitutes promising targets for TAM repolarization in anti-cancer therapies. Targeting metabolic pathways which underpin the polarization and survival of TAMs in the TME could overcome immunosuppression by reducing macrophage recruitment, depleting TAMs, and inducing the pro-inflammatory activation of TAMs [159–161].

Current approaches based on M2-like TAM depletion or M1 polarization suffer from limited efficacy due to the existence of resistance mechanisms [162], the re-infiltration of macrophages after therapy [163], and the presence of other immunosuppressive cells such as regulatory T cells [164]. Besides, different immune cells may reply on similar metabolic pathways to support their activities in the TME [31, 165]. Thus, it seems inevitable that modulation of core metabolic processes may exert undesirable immunological effects on other immune cells in the TME. The presence of different cytokines and the interaction of TAMs with different cell types add another layer of complexity in TAM functional polarization in the TME [166, 167]. The key determinants in macrophage differentiation in the TME need to be clarified. The understanding of the most critical metabolic pathway underlying TAM polarization is conducive to more precisely targeting TAM metabolism without favoring tumor growth.

On the other hand, whether TAM-derived metabolites are implicated in the progression of tumor cells remains largely unknown. Further, how TAMs undergo temporal and spatial metabolic changes in tumor progression? The potential metabolic rewiring of TAMs at different cancer stages (primary and metastatic tumors) may require more tailored strategies for TAM repolarization [168]. Moreover, TAM metabolic profile changes before and after chemotherapy and immunotherapy may be also relevant to the therapeutic response of a patient [169]. In an era of single cell omics, the heterogeneity of TAMs in different cancers has begun to be unveiled [170]. The characterization of diverse TAM subpopulations can provide novel insights into the functional plasticity of TAMs in the TME. Future works are warranted to decipher the metabolic underpinnings of different functional groups of TAMs.

Although most metabolic reprogramming strategies are in the preclinical stage and the therapeutic potential in cancer patients remains to be validated, they provide opportunities to reshape the immune microenvironment in tumors. We surmise that some of these interventions could be integrated with the current immunotherapy to boost anti-tumor immunity in the TME.

## Data Availability

Not applicable.

## Abbreviations

α-KG: α-Ketoglutarate
2-DG: 2-Deoxy-d-glucose
ACLY: ATP-citrate lyase
ACOD1: Aconitate decarboxylase 1
AHR: Aryl hydrocarbon receptor
AKT: Protein kinase B
AMPK: Adenosine 5'-monophosphate–activated protein kinase
ARG1: Arginase 1
CCL22: C–C motif chemokine ligand 22
CD39: Ectonucleoside triphosphate diphosphohydrolase 1
CD73: Ecto-5ʹ-nucleotidase
Cpt1a: Carnitine palmitoyltransferase 1A
CTL: Cytotoxic T cell
DDIT4: DNA Damage Inducible Transcript 4
ECAR: Extracellular acidification rate
ER: Endoplasmic reticulum
ERS: Endoplasmic reticulum stress
FAO: Fatty acid oxidation
FCR: Fc receptors
GLUD1: Glutamate dehydrogenase 1
GLS: Glutaminase
GS: Glutamine synthetase
HIF: Hypoxia inducible factor
IDH: Isocitrate dehydrogenase
IDO: Indoleamine-2,3-dioxygenase
IFN-γ: Interferon gamma
IL-13: Interleukin-13
IL-4: Interleukin-4
iNOS: Inducible nitric oxide synthase
IRE1: Inositol-requiring enzyme 1
IRG1: Aconitate decarboxylase 1
ITA: Itaconate
JAK1: Janus Kinase 1
JMJD3: Jumonji-C-domain-containing histone demethylase 3
KYN: Kynurenine
LDHA: Lactate dehydrogenase A
LPS: Lipopolysaccharide
M-CSF: Macrophage colony stimulating factor
MCP-1: Monocyte chemoattractant protein-1
MDH1: Malate dehydrogenase 1
ME1: Malic enzyme 1
MSO: Methionine sulfoximine
MSR1: Macrophage scavenger receptor 1
mTORC2: Mammalian target of rapamycin complex 2
NAD: Nicotinamide adenine dinucleotide
NADH: Nicotinamide adenine dinucleotide hydrogen
NADPH: Nicotinamide adenine dinucleotide phosphate
NF-κB: Nuclear factor-κB
NO: Nitric oxide
NOXs: NADPH oxidases
OCR: Oxygen consumption rate
OXPHOS: Oxidative phosphorylation
p70S6K: P70 ribosomal protein S6 kinase
PDAC: Pancreatic ductal adenocarcinoma
PDK1: Pyruvate dehydrogenase kinase isozyme 1
PERK: Protein kinase RNA-like ER kinase
PGC1α: Proliferator-activated receptor gamma coactivator 1-alpha
PHD: Prolyl hydroxylase
PI3K: Phosphatidyl inositol 3-kinase
PIP2: Phosphatidylinositol 4,5-bisphosphate
PIP3: Phosphatidylinositol 3,4,5-triphosphate
PKM2: Pyruvate kinase 2
PPARs: Peroxisome proliferator activation receptors
PPP: Pentose phosphate pathway
RBM4: RNA-binding motif 4
REDD1: DNA Damage Inducible Transcript 4
RIPK3: Receptor-interacting protein kinase 3
ROS: Reactive oxygen species
SDH: Succinate dehydrogenase
STAT1: Signal transducer and activator of transcription 1
SUCNR1: Succinate receptor
TAMs: Tumor-associated macrophages
TCA: Tricarboxylic acid
TDO: Tryptophan 2,3-dioxygenase
TLR9: Toll-like receptor 9
TLRs: Toll-like receptors
TME: Tumor microenvironment
Tregs: Regulatory T cell
VEGF: Vascular endothelial growth factor
VEGFC: Vascular endothelial growth factor C
XBP1: X-box binding protein 1
